# Morphological variability and genetic diversity in *Carex buxbaumii* and *Carex hartmaniorum* (Cyperaceae) populations

**DOI:** 10.7717/peerj.11372

**Published:** 2021-05-11

**Authors:** Helena Więcław, Magdalena Szenejko, Thea Kull, Zofia Sotek, Ewa Rębacz-Maron, Jacob Koopman

**Affiliations:** 1 Institute of Marine and Environmental Sciences, University of Szczecin, Szczecin, Poland; 2 Molecular Biology and Biotechnology Center, University of Szczecin, Szczecin, Poland; 3 Institute of Agricultural and Environmental Sciences, Estonian University of Life Sciences, Tartu, Estonia; 4 Institute of Biology, University of Szczecin, Szczecin, Poland; 5 Unaffiliated, Choszczno, Poland

**Keywords:** Discriminant function analysis, Genetic affinity, Intra- and interpopulation variability, ISSR polymorphism, Phenotypic plasticity, *Racemosae*

## Abstract

**Background:**

*Carex buxbaumii* and *C*.* hartmaniorum* are sister species of the clade *Papilliferae* within the monophyletic section *Racemosae*. An unambiguous identification of these species is relatively difficult due to the interspecific continuum of some morphological characters as well as the intraspecific variability. The study was aimed at determining the range of variability, both morphological and genetic, within and between these two closely related and similar species.

**Methods:**

The sedges were collected during botanical expeditions to Armenia, Estonia, the Netherlands, and Poland. The morphological separation of the two species and their populations was tested using the Discriminant Function Analysis (DFA). The genetic variability of the 19 *Carex* populations was assessed in the presence of eight Inter Simple Sequence Repeat (ISSR) primers.

**Results:**

Results of the study indicate a considerable genetic affinity between the two sedge species (mean Si = 0.619). However, the populations of* C*.* hartmaniorum* are, morphologically and genetically, more homogenous than the populations of *C*. *buxbaumii*. Compared to *C*. *hartmaniorum, C. buxbaumii* usually has wider leaf blades, a shorter inflorescence, a lower number of spikes which are shorter, but wider, and longer bracts and utricles. The AMOVA showed a larger variation between the populations of *C. buxbaumii*, representing 25.65% of the total variation in the taxon. Two populations of *C*. *buxbaumii* (from Poland and Estonia) are separated from the remaining populations, both genetically and morphologically; their individuals show shorter utricles and glumes, compared to the typical specimens of *C*. *buxbaumii*, and correspond with the morphology of putative infraspecific taxa described by Cajander (var. *brevisquamosa* and var. *confusa*).

**Conclusions:**

The taxonomic status of the putative infraspecific taxa within * C*. *buxbaumii* requires further studies throughout the distribution range of *C*. *buxbaumii*, addressing habitats, morphology and genetics (including a chromosome count or a combination of different genetic methods), particularly as the variability in *C*. *buxbaumii* may be associated with the species’ polyploid origin.

## Introduction

*Carex buxbaumii* Wahlenb. and *C*. *hartmaniorum* A.Cajander belong to the section *Racemosae* G.Don [synonyms: sect. *Microrhynchae* (Drej.) L.H.Bailey, sect. *Atratae* (Heuff.) Christ, sect. *Loxaniza* (Raf.) V.I.Krecz.] which counts over 60 species worldwide, growing mainly in wetlands but also on dry plains in the Arctic and alpine tundra of North America and Eurasia, rarely in South America (e.g., Egorova, 1999; Murray, 2002; Liang & Koyama, 2010; Reznicek & Murray, 2013; Koopman, 2015; Jiménez-Mejías et al., 2018).

*Carex buxbaumii* has a circumpolar northern distribution, centred in the boreal to arctic zones. It occurs mainly in northern Europe and Asia (it is scattered in the central and southern part of both continents) as well as in North America and the northern part of South America (Govaerts et al., 2021). *Carex buxbaumii* grows mainly in calcareous, moist to wet meadows, in marshes and fens, but is known also from wet slopes in the subalpine belt (Sotek, 2006; Kaplan et al., 2018; Sienkiewicz-Paderewska et al., 2019).

The main area of *C*. *hartmaniorum* extends from Europe to central Asia (Govaerts et al., 2021); its northward boundary is southern Scandinavia, the southward boundary running through France and northern Italy, the Balkans and the Caucasus (Guglielmetto Mugion & Rivella, 1995; Hájek, Hájková & Apostolova, 2005; Galtier & Guillerme, 2011; Koopman et al., 2015). This sedge grows mainly in wet meadows and base-rich fens (e.g., Sotek, 2008; Kaplan et al., 2018).

*Carex buxbaumii* was described in 1803 from Sweden, whereas *Carex hartmaniorum* was separated from *C*. *buxbaumii* (*C*. *polygama* agg. *sensu* A.Cajander) in 1935 (Cajander, 1935). Thus, for more than 130 years, they were treated as a single species. Owing to the morphological similarity, Egorova (1985) assigned them to the subsection *Papilliferae* T.V.Egorova within the section *Racemosae*. Recent morphological studies and molecular phylogenetic analyses have confirmed the affinity between *C*. *buxbaumii*, *C*. *hartmaniorum* and *C*. *adelostoma* V.I.Krecz., and indicated their close relationship with *C*. *holostoma* Drej. (Gebauer, Röser & Hoffmann, 2015; Massatti, Reznicek & Knowles, 2016; Więcław et al., 2017; Roalson et al., 2020). *Carex buxbaumii* and *C*. *hartmaniorum* are sister species and, together with the species listed above, are at present assigned to the clade *Papilliferae* within the monophyletic section *Racemosae* (Global Carex Group, 2016; Massatti, Reznicek & Knowles, 2016; Martín-Bravo et al., 2019).

*Carex buxbaumii* and *C*. *hartmaniorum* are both characterised by long creeping rhizomes and densely papillose utricles with a very short bifid beak. In addition, the two sedges usually show a gynaecandrous terminal spike and 2–6 lateral female spikes. The lowest bract is leaf-like. The female glumes are ovate-lanceolate with an acuminate or aristate apex (Kükenthal, 1909; Chater, 1980; Egorova, 1999). Differences between these species are fairly subtle, and dimensions of numerous morphological characters overlap, which considerably hampers an unambiguous identification. However, the inflorescences of *C*. *buxbaumii* are usually shorter, spikes are shorter and wider, and utricles are longer than those in *C*. *hartmaniorum* (Więcław et al., 2017). Moreover, a distinct intraspecific variability in these two species can be observed, particularly within *C. buxbaumii* with its two varieties, var. *confusa* and var. *brevisquamosa*, described by Cajander (1935). Establishing the range of morphological variability within and between such closely related species will make it possible to single out the taxonomically important characters, useful for the identification. Morphological characters with which organisms are identified and described are a major practical criterion used in plant systematics as numerous systematic descriptions are based on morphological data (MacLeod, 2002). However, using morphological data alone for species delimitation involves a number of drawbacks stemming from intraspecific morphological variability or small morphological differences between closely related species (e.g., Whittall et al., 2004; Duminil & Di Michele, 2009). Combining morphological studies with genetic assays provides insights into speciation processes and is of a fundamental importance for taxonomy, particularly at species level. Studies on genetic variability among closely related species, the taxonomic distinctness of which is usually obliterated in their morphology, allow to assess the extent of inter- and intraspecific variation and to explore relationships between the species studied (e.g., Schönswetter et al., 2009; Szczepaniak & Cieślak, 2011; Hurry, Walsh & Murphy, 2012; Mosaferi et al., 2015; Janković et al., 2019). Assessment of the extent of morphological and genetic variability are important for identifying taxonomic boundaries, developing natural classification systems, and for reconstructing phylogenies (e.g., Giulietti et al., 2012; Flores-Rentería et al., 2013; Lissambou et al., 2019).

Because of the interspecific continuum in some morphological characters, observed in *C*. *buxbaumii* and *C*. *hartmaniorum*, which frequently renders an unambiguous identification of specimens impossible, as well as due to the intraspecific variability, the present study was aimed at assessing the extent of morphological and genetic variability within and between those two closely related and similar species.

## Materials & Methods

### Plant material and specimen collection

**Figure 1:**
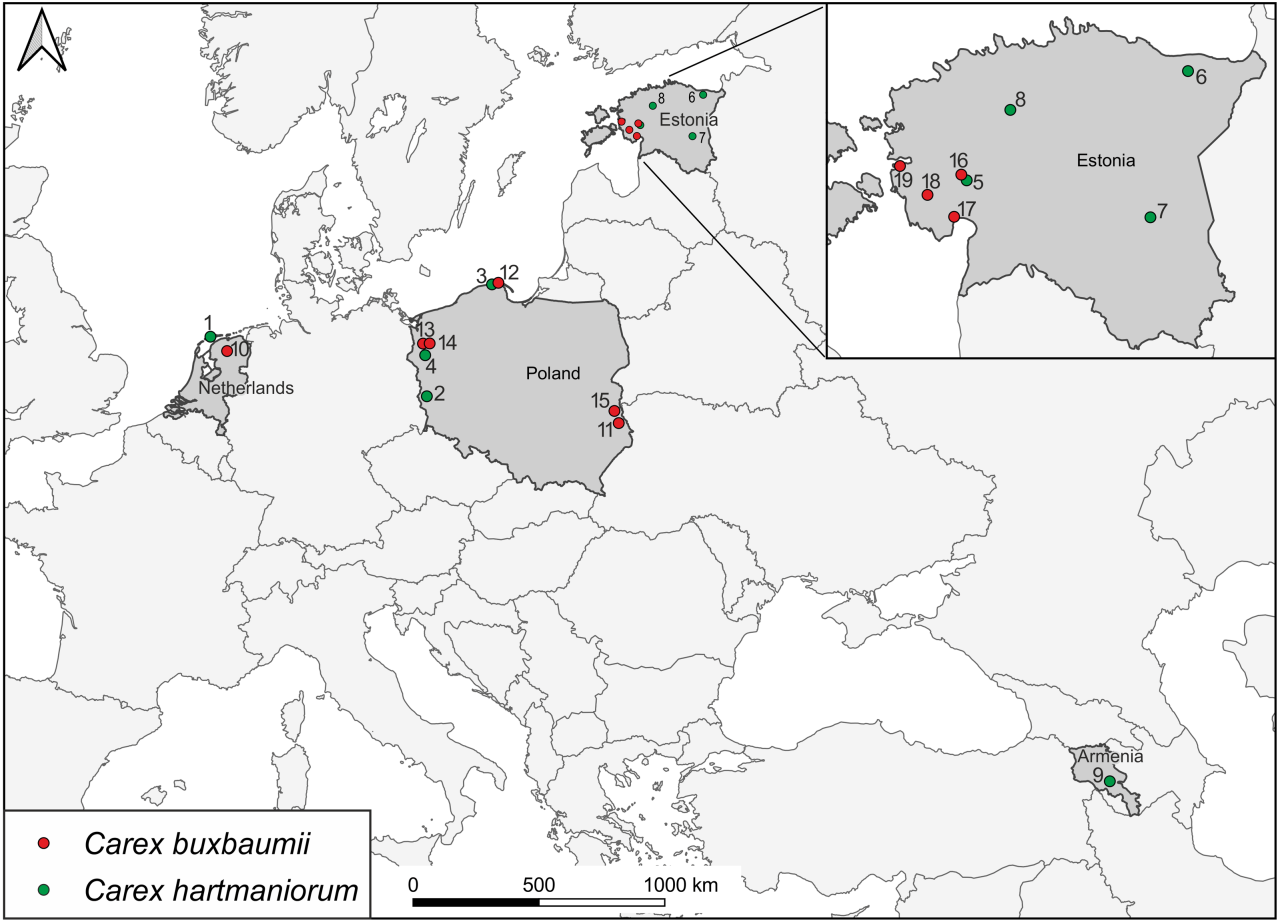
Locations of *Carex buxbaumii* and *C*. *hartmaniorum* sites sampled in this study. The populations are numbered according to Table 1.

Sedges were collected during botanical expeditions to Armenia, Estonia, the Netherlands, and Poland (Fig. 1; Table 1). A total of 300 specimens from 15 populations were examined morphologically; there were 160 specimens of *C*. *buxbaumii*, with eight populations composed 20 specimens each, and 140 specimens of *C*. *hartmaniorum*, with seven populations, 20 specimens each. Molecular assays focused on 19 populations, with three specimens each (a total of 57 specimens). Samples within a population were collected 2–20 m apart from each other to reduce the chance of collecting individuals from the same clone. The samples were collected from an area usually 2–8 ha in size. In the Netherlands, *C*. *buxbaumii* (population 10) individuals were highly scattered, for which reason the specimens were collected from a larger area (more than 10 ha).

**Table 1:** Information about the stands of *Carex hartmaniorum* and *C. buxbaumii*.

Population	Location/habitat	Coordinates	Collector
*Carex hartmaniorum*			
1^*^	Netherlands, Terschelling, Noordsvaarder, Fryslân/wet dune valley grassland	53.42277 05.40696	H. Więcław & J. Koopman
2^*^	Poland, Górzyn/wet meadow	51.82214 14.99236	H. Więcław & J. Koopman
3	Poland, Piaśnica/calcareous meadow	54.82274 18.06363	H. Więcław & J. Koopman
4^*^	Poland, Otanów, E of jezioro Chłop/calcareous meadow	52.98928 14.90003	J. Koopman & H. Więcław
5^*^	Estonia, Langerma/edge of a wet fen	58.64657 24.41587	T. Kull
6	Estonia, Vitsiku/pasture, actively in use, calcareous, moderately moist	59.33768 27.23209	T. Kull
7^*^	Estonia, Raadi/paludified meadow	58.38949 26.76064	T. Kull
8^*^	Estonia, Mahtra/paludified meadow	59.08855 25.00219	T. Kull
9^*^	Armenia, c. 12 km SSW of Martuni, W of road to Selim pass/wetland in river valley	40.03567 45.24250	J. Koopman & H. Więcław
*Carex buxbaumii*			
10^*^	Netherlands, Wijnjeterper Skar, Fryslân/ wet meadow	53.05855 06.16415	H. Więcław & J. Koopman
11^*^	Poland, S of Chełm, Krzywiczki/calcareous meadow	51.08283 23.49236	H. Więcław & J. Koopman
12	Poland, Piaśnica/calcareous meadow	54.82274 18.06363	H. Więcław & J. Koopman
13^*^	Poland, E of Giżyn, SW of Jezioro Miedwie/wet meadow	53.22509 14.86649	J. Koopman & H. Więcław
14^*^	Poland, E of Giżyn, SW of Jezioro Miedwie/calcareous meadow	53.22516 14.86598	J. Koopman & H. Więcław
15	Poland, Krowie Bagno/calcareous fen	51.41917 23.30222	P. Sugier & Z. Sotek
16^*^	Estonia, Langerma/edge of a wet fen	58.64676 24.41528	T. Kull
17^*^	Estonia, Audru/paludified meadow, on the edge of a gravel road	58.39301 24.29674	T. Kull
18^*^	Estonia, Peantse/calcareous fen	58.53658 23.96249	T. Kull
19^*^	Estonia, Metsküla/calcareous meadow, temporarily wet	58.72520 23.61733	T. Kull

**Notes.**

* Populations used in morphological analyzes are marked with an asterisk.

The herbarium material was deposited at the Herbarium Stetinensis (SZUB) and the Herbarium of Estonian University of Life Sciences (TAA).

### Nomenclature

The nomenclature used follows the WCSP (Govaerts et al., 2021). The name *Carex hartmanii* does not comply with the International Code of Nomenclature for algae, fungi, and plants (Turland et al., 2018). Cajander (1935), introducing into the botanical nomenclature the name *Carex hartmanii*, wished to honour both Carl Johan Hartman (father) and Carl Hartman (son). Thus, in accordance with article 60.8(b) and Note 4 of the International Code of Nomenclature (Turland et al., 2018), the correct name is *Carex hartmaniorum* (see Buttler, 2017).

### Morphological characters

Twelve quantitative morphological characters (Table 2) were measured. The length of utricles, beaks and glumes was measured to 0.01 mm under a stereo microscope. Five utricles and glumes from the middle part of a spike of each specimen were isolated for measurements and the measurements averaged. Utricles from the central part of a spike are regarded as least variable and are most often used in biometric studies (e.g., Więcław, 2014). Other measurements were taken with a Vernier callipers to 0.05 cm (inflorescence length, uppermost and lowest spikes size, lowest bract length and leaf width) and with a ruler to 0.1 cm (culm height).

**Table 2:** Quantitative characters used in morphological analyses.

Characters	Abbreviation
Culm height (cm)	CH
Leaf width (cm)	LW
Bract length (cm)	BL
Inflorescence length (cm)	IL
Number of spikes (no)	NS
Uppermost spike length (cm)	USL
Uppermost spike width (cm)	USW
Lowest spike length (cm)	LSL
Lowest spike width (cm)	LSW
Utricle length (mm)	UL
Utricle beak length (mm)	UBL
Glume length (mm)	GL

### DNA isolation

The genome DNA was isolated, using a NOVABEADS Plant DNA Standard Kit II (Novazym, Poznań, Poland), from dried leaves of three plants from each population. The magnetic bead-based DNA extraction method was applied and the monocots protocol was followed (PROTOCOL, 2021). The concentration and quality of the DNA extracts were assessed electrophoretically and with a NanoDrop 2000C (Thermo Fisher Scientific, USA) spectrophotometer.

### ISSR method

The ISSR (Inter Simple Sequence Repeat) method is based on inter-microsatellite sequence polymorphism and has been frequently used to assess genetic variability of different plant species, including closely related *Carex* taxa (Liu, Wei & Dong, 2009; Korpelainen et al., 2010; Ning et al., 2014; Szenejko, Śmietana & Stępień, 2016).

The ISSR analysis was run according to a modified method of Ziętkiewicz, Rafalski & Labuda (1994). A total of 80 microsatellite primers, manufactured by Genomed S.A. (Warsaw, Poland) and Oligo IBB PAN (Warsaw, Poland), were tested. Eventually, the ISSR polymorphism assay involved 8 primers (Table 3). The reaction mix, 20 µl in volume, included: 2x Phire Green Hot Start II PCR Master Mix (Thermo Fisher Scientific), 0.8 µM of the primer, water, and 50 ng DNA. Amplification was performed in a T100™ Thermal Cycler (Bio-Rad, California, USA) thermocycler, in 40 cycles, with the following thermal profile: initial denaturation at 98 °C for 5 min, then 98  °C for 5 s, 52–53 °C (depending on the type of primer, see Table 3) for 5 s, 72  °C for 20 s, and the final elongation at 72  °C for 1 min. The optimal temperature of primer binding (Ta) was adjusted for each ISSR primer individually, using the thermocycler gradient. The ISSR amplification products obtained were separated on a 2.5% agarose gel with a SimplySafe™ dye (5 µg/ml) (EURx, Gdańsk, Poland) in the TAE buffer, in the presence of the NZYDNA Ladder VII (Nzytech Genes&Enzymes, Lisbon, Portugal) mass standard, for 7 h. Visualisation, documentation and analysis of the results was carried out using a Gel Doc™ XR+ kit and the Image Lab™ Software 4.0 (Bio-Rad).

**Table 3:** Characteristics of the eight primers used in this study.

Primer	Microsatelite	Sequence 5′–3′	Ta
810	(GA)_8_T	GAGAGAGAGAGAGAGAT	52
811	(GA)_8_C	GAGAGAGAGAGAGAGAC	52
813	(CT)_8_T	CTCTCTCTCTCTCTCTT	53
834	(AG)_8_YT	AGAGAGAGAGAGAGAGYT	52
840	(GA)_8_CT	GAGAGAGAGAGAGAGACT	53
842	(GA)_8_YG	GAGAGAGAGAGAGAGAYG	53
888	BDB(CA)_7_	BDBCACACACACACACA	53
M21	(TC)_8_C	TCTCTCTCTCTCTCTCC	53

**Notes.**

Eight microsatellite primers were used for the ISSR-PCR reaction. Half of them were dinucleotide repeat sequences (GA), anchored at the end of 3’. Y - T or C; B - C, G or T; D - A, G or T; Ta - annealing temperatures (°C).

### Data analysis

Since the distribution of most datasets departed from normality (Shapiro–Wilk’s test), the non-parametric Mann–Whitney U test was used to examine whether morphological differences between *C*. *buxbaumii* and *C*. *hartmaniorum* were significant. The Kruskal-Wallis test and Dunn’s test of multiple comparisons were used to pinpoint those populations differing significantly from others. Morphological separation of the specimens was tested using Discriminant Function Analysis (DFA). The data used in DFA were standardized so that each variable would have a mean of 0 and a standard deviation of 1. Calculations were performed using the software package Statistica v. 13.1 for Windows (StatSoft, Inc., 2013).

For each sedge population studied, the number (P) and per cent contribution (%P) of polymorphic loci were computed, as were two indices of genotypic diversity: the Shannon index (I) and the gene population diversity (h; Nei, 1973). The Shannon index value was calculated according to the formula I = − ∑ *pi* log_2_pi, while the gene population diversity index is based on the formula h =1 −∑pi^2^, where *p*_*i*_ is the frequency of the *i* th allele. The value of the Polymorphism Information Content (PIC) for a dominant marker system was calculated as PIC = 1 - p2 - q2, where *p* is band frequency and *q* is no-band frequency (Ghislain et al., 1999). The value of the Assay Efficiency Index (AEI), which indicates an average number of polymorphic products identified in the presence of a single primer, was also calculated (Pejic, Ajmone-Marsan & Morgante, 1998). The intra- and interpopulation genetic variability of *C*. *hartmaniorum* and *C*. *buxbaumii* was analysed using the Analysis of Molecular Variance (AMOVA) run with the aid of the GenAIEx 6.5 software (Peakall & Smouse, 2012). A genetic similarity matrix was constructed based on the Dice similarity index [Si; according to Dice (1945) following Nei & Li (1979)] with the FreeTree software (Hampl, Pavliček & Flegr, 2001; Pavlíček, Hrdá & Flegr, 1999). The following formula was used: Si = 2Nij/(Ni+Nj), where Nij is the number of bands present in both genotypes *i* and *j*, Ni is the number of bands present in genotype *i*, and Nj is the number of bands present in genotype *j*. The genetic similarity matrix obtained served to run two multivariate statistical analyses, both based on Euclidean distances: the hierarchical clustering (UPGMA) and the Principal Component Analysis (PCA). As the PCA is relatively objective and provides a reasonable indication of relationships, it was used to confirm the similarity of the grouping obtained with the UPGMA dendrogram. These two analyses were carried out using the Statistica v. 13.1 for Windows software package (StatSoft, Inc., 2013).

## Results

### Morphological variability

**Figure 2:**
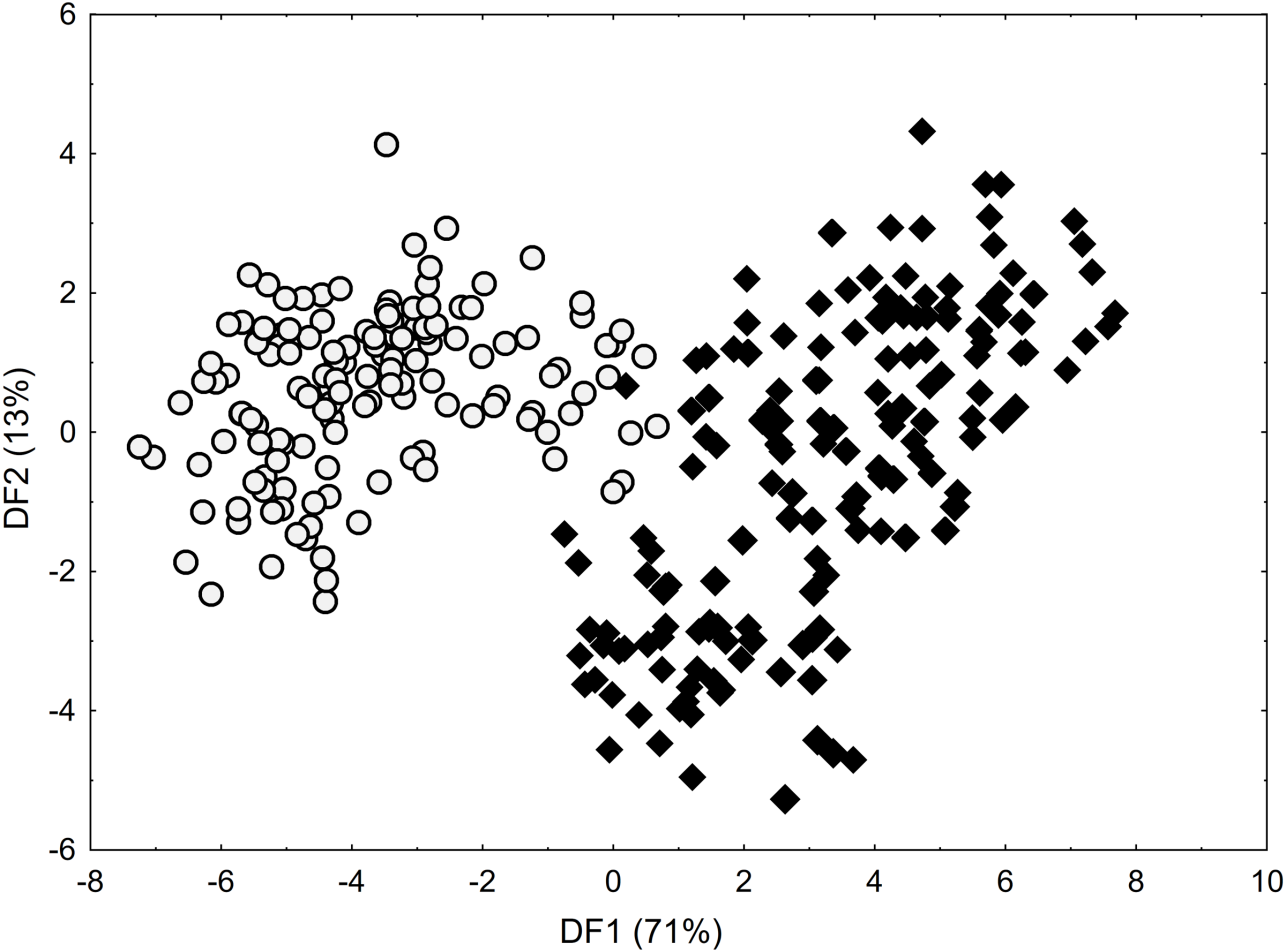
Discriminant scores for individuals of *Carex buxbaumii* (diamond) and *C*. *hartmaniorum* (circle). Loadings for the first discriminant axis DF1 (only absolute values > 0.5): IL (inflorescence length) = − 0.61, BL (bract length) = 0.62, and UL (utricle length) = 0.60. Loadings for the second discriminant axis DF2 (only absolute values > 0.5): CH (culm height) = − 0.71 and UL (utricle length) = − 0.55.

As shown by the Mann–Whitney *U* test, *C*. *buxbaumii* and *C*. *hartmaniorum* differ significantly in terms of nine characters analysed (Table S1). Compared to *C*. *hartmaniorum, C. buxbaumii* usually has wider leaf blades, a shorter inflorescence, a lower number of spikes which are shorter, but wider, and longer bracts and utricles (Table S2; Figs. S1–S3). The DFA on the entire data set (Fig. 2) produced no clear separation between the two species, but along the first discriminant function (axis), most of the *C*. *buxbaumii* specimens were placed on the right-hand side of the diagram, while most of the *C*. *hartmaniorum* ones being placed on the left-hand side. *Carex hartmaniorum* formed a fairly compact group in the ordination space, while the *C*. *buxbaumii* individuals being more scattered. The strongest effect on the first discriminant function was exerted by the inflorescence length (IL), bract length (BL), and utricle length (UL), whereas the second function is determined mostly by the culm height (CH) and utricle length (UL). The first two discriminant functions collectively explain 84% of the variance (71 and 13%, respectively) (Fig. 2).

**Figure 3:**
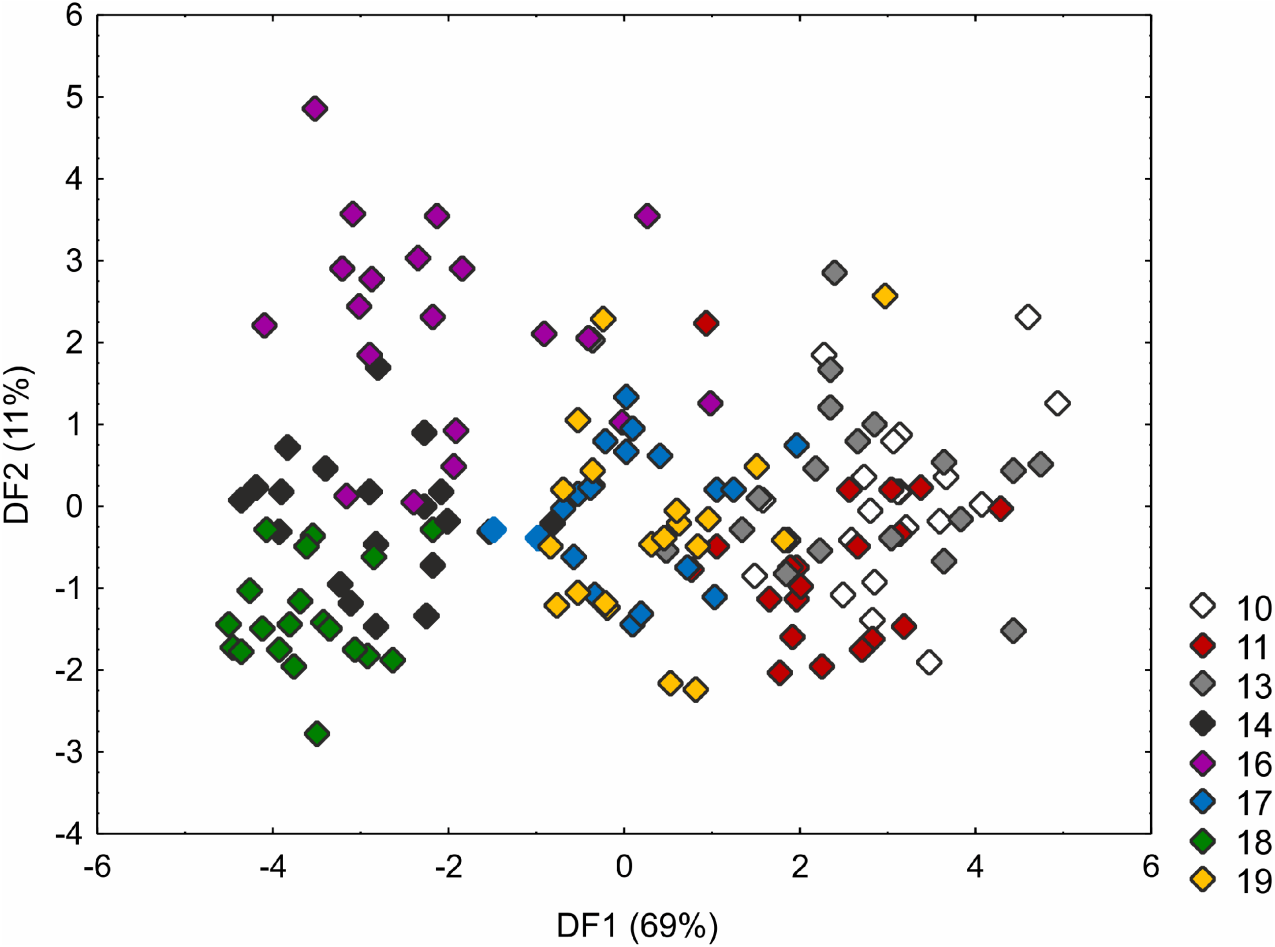
Discriminant scores for individuals of *Carex buxbaumii* populations. Loadings for the first discriminant axis DF1 (only absolute values > 0.5): CH (culm height) = − 0.60, IL (inflorescence length) = − 1.14, BL (bract length) = 0.82, and GL (glume length) = 0.60. Loadings for the second discriminant axis DF2 (only absolute values > 0.5): IL (inflorescence length) = 1.04 and BL (bract length) = − 0.92. The populations are numbered according to Table 1; 10—population from the Netherlands, 11, 13 and 14—populations from Poland, 16, 17, 18 and 19—populations from Estonia.

The Kruskal–Wallis test and the post-hoc Dunn’s test of multiple comparisons identified those *C*. *buxbaumii* populations which were significantly different from others studied (Table S3). The Polish (number 14) and Estonian (16 and 18) populations are clearly morphologically different, which is also visible in the distribution of the specimens in the DFA space (Fig. 3). Specimens from those populations were placed in the left-hand part of the diagram, while individuals from populations 10, 11 and 13 are scattered in the opposite part along the first axis, specimens of populations 17 and 19 being located in the middle part of the axis. The first discriminant function is defined by the culm height (CH), inflorescence length (IL), bract length (BL) and glume length (GL) (Fig. 3). Specimens from populations 14, 16 and 18 were usually higher, their inflorescences were longer and the bracts and glumes shorter (Fig. S1). Populations 16 and 18 were found to grow at fairly shaded sites in a fen with up to 50 and 30% bush cover, respectively. Population 14 grew on a meadow overgrown by *Phragmites australis*. The second axis, formed mostly by the inflorescence length (IL) and bract length (BL) separates most of the specimens of population 16 from populations 14 and 18 (Fig. 3). Specimens of population 16 showed relatively longer inflorescences and bracts (Fig. S1). The first two axes combined explain 80% of the variance in the data (69 and 11%, respectively; Fig. 3).

**Figure 4:**
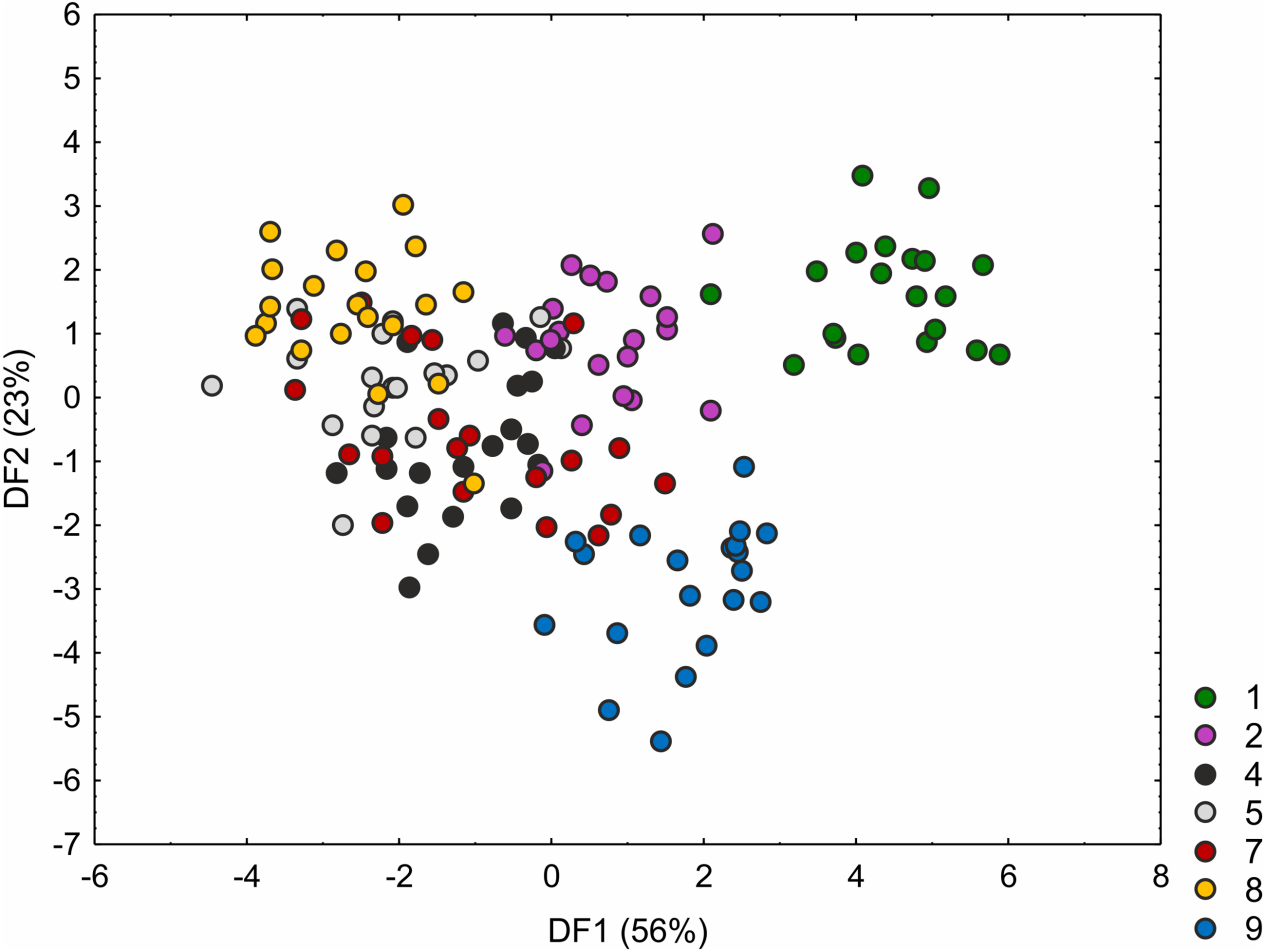
Discriminant scores for individuals of *Carex hartmaniorum* populations. Loadings for the first discriminant axis DF1 (only absolute values > 0.5): CH (culm height) = − 0.68, LSW (lowest spike width) = 0.51, and UL (utricle length) = 0.65. Loadings for the second discriminant axis DF2 (only absolute values > 0.5): LW (leaf width) = − 0.57, IL (inflorescence length) = 1.05, USL (uppermost spike length) = 0.63, BL (bract length) = − 1.47, and UL (utricle length) = 0.62. The populations are numbered according to Table 1; 1—population from the Netherlands, 2 and 4—populations from Poland, 5, 7 and 8—populations from Estonia, 9—population from Armenia.

The *C*. *hartmaniorum* populations were found to differ significantly from each other in all the morphological characters analysed (see results of the Kruskal–Wallis test and the post-hoc Dunn’s test in Table S4). In the ordination space, along the first axis, the Dutch specimens (population 1) form a separate group (Fig. 4). The specimens of population 1 showed relatively long utricles and wide spikes (Figs. S2 and S3). The strongest effects on the first axis are exerted by the utricle length (UL), the lowest spike width (LSW), and the culm height (CH). The second discriminant function is defined by the leaf width (LW), the inflorescence length (IL), the uppermost spike length (USL), the bract length (BL), and the utricle length (UL) (Fig. 4). Most specimens of the Armenian population (population 9) are located in the lower part of the diagram (along the second axis); those individuals were generally shorter, their leaf blades being usually narrower and the spikes shorter (Figs. S1 and S2). The first two discriminant functions explain jointly 79% of the total variance in the data (59 and 20%, respectively; Fig. 4).

### ISSR polymorphism and basis genetic diversity parameters

A total of 128 DNA amplification products were obtained in the presence of 8 ISSR primers; the products showed a wide size range, from 2500 bp (in the presence of primer 840) to 180 bp (primer 813), 87.5% of the products being polymorphic (Table 4). The microsatellite primers used in the assays made it possible to identify 22 unique products, characteristic of a single population only. More such products were generated for *C. buxbaumii*: a total of 16 specific amplicons for all the genotypes, except for two Polish populations (13 and 15).

**Table 4:** Amplified products obtained with ISSR primers for *C. hartmaniorum* and *C. buxbaumii* populations.

Primer	Size range (bp)	Total of number products	PB	PPB(%)	UB	PIC
810	2,172–258	12	12	100.00	4	0.280
811	1,624–342	17	15	88.24	2	0.255
813	1,455–180	9	7	77.78	3	0.184
834	2,500–364	16	14	87.50	3	0.239
840	2,058–265	23	22	95.65	2	0.261
842	1,449–260	20	18	90.00	1	0.272
888	1,141–182	15	11	73.33	4	0.166
M21	1,688–395	16	14	87.50	3	0.174
Total (mean per primer)	2,500–180 (1,761–281)	128 (16.0)	113 (14.1)	– (87.500)	22 (2.8)	– (0.236)

**Notes.**

PB number of polymorphic bands PPB the percentage of polymorphic bands UB number of unique bands PIC value of Polymorphism Information Content

The Amplification Effectiveness Index (AEI), which informs on the average number of polymorphic amplification products obtained in the presence of a single primer, was about 14 (Table 4). The highest number of such products was detected in the presence of microsatellites 810, 840, 842 and 811. They were dinucleotide replicate sequences (GA)_8_, anchored at the 3′  ending. Their efficiency in the detection of polymorphism among the sedges studied was confirmed by PIC values, which were higher than those obtained for other primers. The average PIC for all the sedge populations studied was not overly high (0.236). The least-informative ISSR starters were 888, M21 and 813, the PIC of which did not exceed 0.2.

A higher number of polymorphic loci (about 64%) was identified in the *C. buxbaumii* populations (Table 5). At the same time, the species showed higher values of h, the genetic variability of the population. This is evidence of a higher within- and between-population genetic variability of *C. buxbaumii* compared to the other, more homogenous, taxon, *C. hartmaniorum*. Regardless of the sedge species, the lowest proportion of polymorphic loci (%P) and the lowest level of genetic variability were typical for the Polish populations, opposed to the populations sampled in the Netherlands, which showed a relatively high variability. Particularly low values were shown by population 3 (%P =46.00; h=0.329) and 14 (% P =43.48; h =0.297). On the other hand, a considerable genetic variability, as determined from allele frequencies, was found in genotypes of *C. buxbaumii*, particularly in the Dutch population 10 (h =0.603) and the Polish population 11 (h =0.596). Within *C. hartmaniorum,* a relatively large h was shown by the Estonian population 5 (h =0.501) and the Armenian population 9 (h =0.489). The sedge populations showed similar values of the Shannon Index (I), which varied within a narrow range from 0.285 (population 10) to 0.366 (population 19). Overall, the genetic variability of both species as expressed by I, was low (0.338) (Tables 5 and 6).

**Table 5:** Summary of genetic diversity estimated using ISSR markers for all populations analyzed in this study.

Species	Countries	P	%P	h	I
*C. hartmaniorum*	All populations (9)	44.78	56.67	0.432	0.341
	The Netherlands	42.00	66.67	0.479	0.331
	Poland	47.33	47.38	0.353	0.353
	Estonia	44.25	60.42	0.466	0.334
	Armenia	42.00	59.52	0.489	0.328
*C. buxbaumii*	All populations (10)	46.70	64.03	0.489	0.335
* *	The Netherlands	35.00	74.29	0.603	0.286
	Poland	43.20	62.86	0.465	0.332
	Estonia	54.00	62.92	0.492	0.352

**Notes.**

P average number of polymorphic loci %P the percentage of polymorphic loci h value of gene diversity of populations I value of Shannon Index

**Table 6:** Evaluation of ISSR polymorphism, genetic variation and genetic similarity for all populations examined in this study.

Parameter/Index	Values
AEI	14.125
%P (range)	60.542
	(43.480–75.560)
PIC (range)	0.236
	(0.166–0.280)
I (range)	0.338
	(0.285–0.366)
h (range)	0.462
	(0.297–0.603)
Si (range)	0.619
	(0.467–0.885)

**Notes.**

The range of parameters value for all analyzed populations were obtained in parentheses.

AEI Assay Efficiency Index %P the percentage of polymorphic loci PIC Polymorphism Information Content I value of Shannon Index h value of gene diversity of populations Si Genetic Similarity Index

The genetic similarity index (Si) calculated for the two species was shown to vary within a relatively wide range, from 0.467 (for two *C. buxbaumii* populations: Dutch population 10 and Estonian population 19) to 0.885 (for two *C. hartmaniorum* populations: Polish population 4 and Estonian population 8) (Table 6). A higher genetic affinity was presented by the *C. hartmaniorum* genotypes. The lowest Si within the taxon (0.656) was found for two Estonian populations (5 and 7). The Dutch population 10 and the Polish population 15 proved the most different from the other ones, with the average Si of 0.619 for the two species.

As shown by AMOVA, the species studied showed small, albeit statistically significant, genetic differences (13.21%), explained in 17.86 and 68.93% by the variability between all the populations and between their specimens, respectively (Table 7). A higher within-species genetic variability was typical of *C. buxbaumii*; the between-populations and within-population variability accounted for 25.65 and 74.35% of the differences, respectively.

**Table 7:** Analysis of molecular variance (AMOVA) calculated for C. hartmaniorum and *C. buxbaumii* populations and individuals.

Taxon	SV	df	SSD	VC	%TV
Two species	Species	1	65.40	1.74	13.21
	Populations	17	273.51	2.34	17.86
	Individuals	38	344.00	9.05	68.93
*C. hartmaniorum*	Populations	8	93.41	1.16	12.45
	Individuals	18	147.33	8.19	87.55
*C. buxbaumii*	Populations	9	180.10	3.39	25.65
	Individuals	20	196.67	9.83	74.35

**Notes.**

SV source of variation df degrees of freedom SSD sum of squares VC variance-component estimates %TV the percentage of total variance

*p* < 0.001 for all cases.

### Cluster and PCA analysis

**Figure 5:**
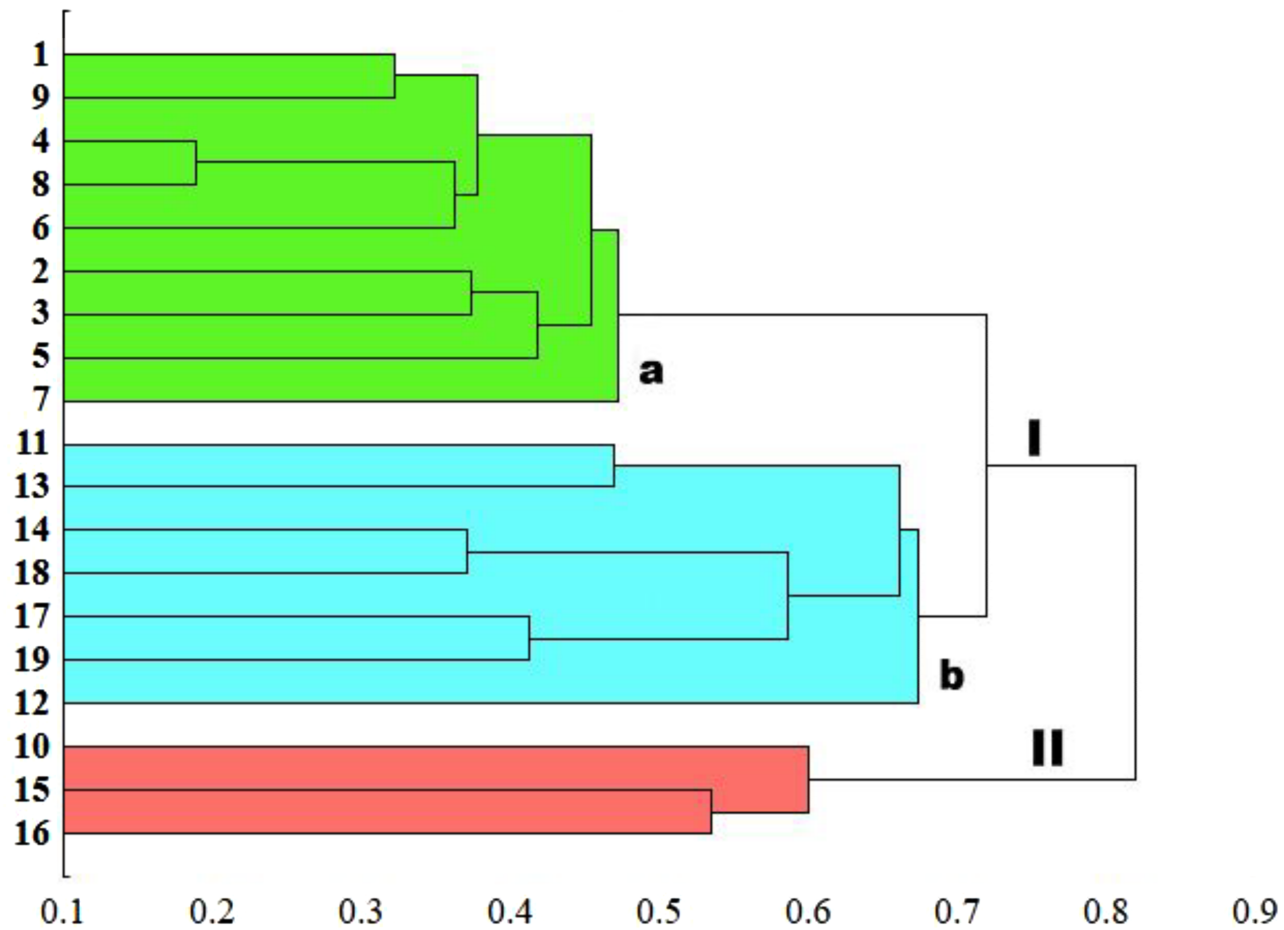
UPGMA dendrogram of genetic similarity of the studied populations of *Carex* obtained on the basis of ISSR markers. The horizontal axis of the dendrogram represents the distance between clusters. The vertical axis represents the populations and clusters. The populations are numbered according to Table 1. I—the first main cluster including both *C*. *buxbaumii* and *C*. *hartmaniorum* populations; a—subcluster containing *C*. *hartmaniorum* populations, b—subcluster containing *C*. *buxbaumii* populations; II—the second main cluster including only *C.* *buxbaumii* populations.

The genetic similarity measure metric developed was used, as a matrix, to construct a relevant UPGMA dendrogram (Fig. 5). Based on the average similarity between all possible pairs of objects, the sedge populations studied were grouped into two asymmetric major clusters (I–II). The first cluster (I) grouped as much as 84.2% of the populations, divided into two separately sub-clusters (a and b). The first sub-cluster (a) contains populations of *C. hartmaniorum* only. Among them, the closest genetic affinity is seen between three pairs of populations: the first pair consists of populations 4 (Poland) and 8 (Estonia), the second contains populations 1 (Netherlands) and 9 (Armenia), the third being formed by populations 2 and 3 (both from Poland). The second sub-cluster (b) contains seven populations of *C. buxbaumii*, including four from Poland (11, 12, 13 and 14) and three from Estonia (17, 18 and 19). In both *Carex* species, the Estonian and Polish genotypes showed a high genetic similarity. The second major cluster (II) groups three populations genetically divergent from the remaining sedges studied: the Dutch population 10 as well as the Polish population 15 and the Estonian population 16, the last two forming a pair in the dendrogram.

**Figure 6:**
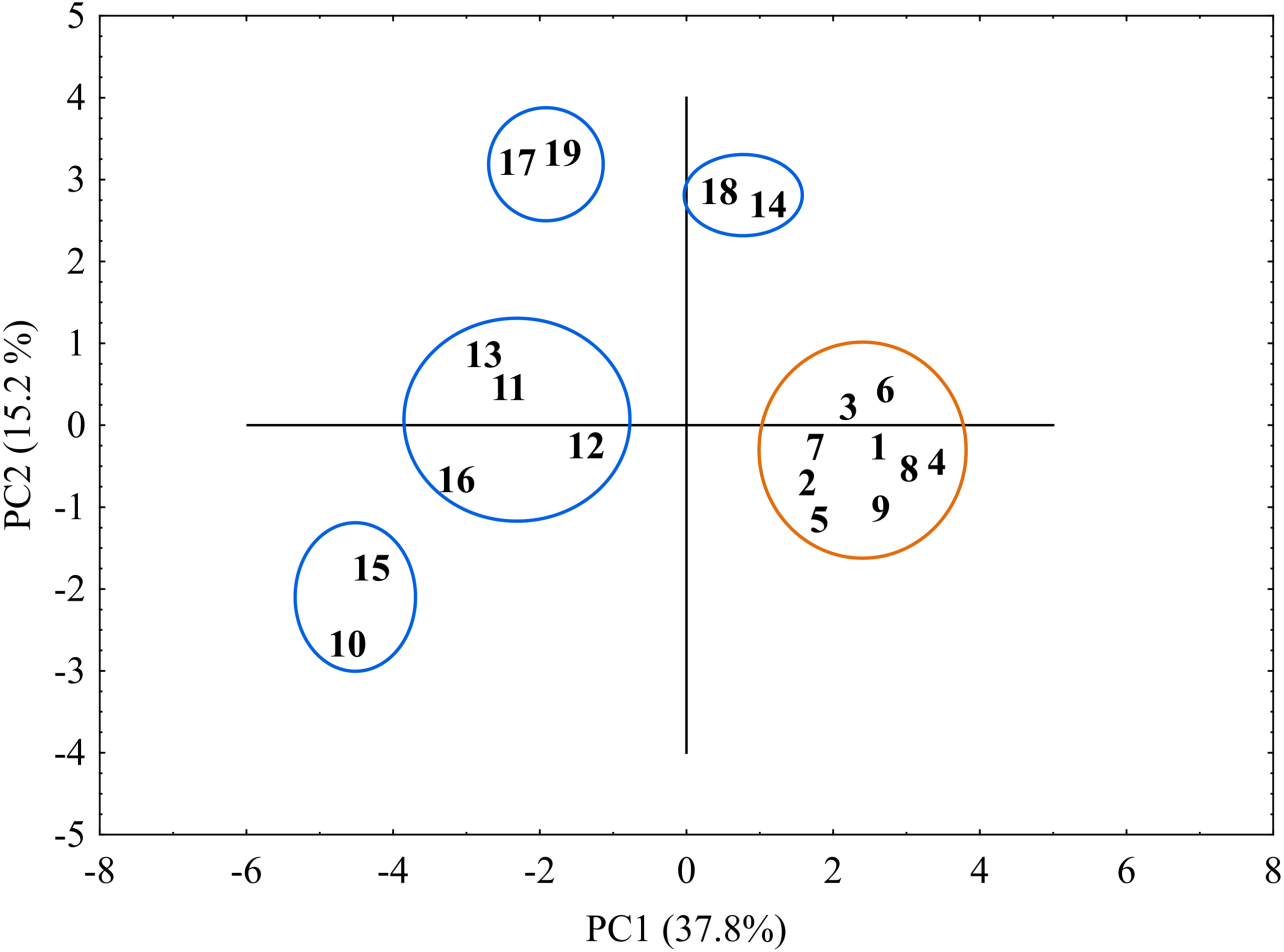
The distribution of the studied populations of *Carex* in two-dimensional space (Principal Component Analysis, PCA). Orange indicates the populations of *C. hartmaniorum*. Blue indicates the populations of *C. buxbaumii*. The populations are numbered according to Table 1.

The genetic similarity matrix was used also to explore the location of the sedge genotypes in a 2-factor space generated by PCA (Fig. 6). The PCA grouping of most of the genotypes studied (17 or 89.5%) proved consistent with the UPGMA clustering. They were arranged on both sides of the origin of the axes and formed five different groups. All the *C. hartmaniorum* specimens grouped together, relatively close to the origin. This is indicative of a low genetic diversity between the *C. hartmaniorum* populations, as opposed to the populations of *C. buxbaumii*, which were divided into four groups. Only one group, formed by the Polish population 14 and the Estonian population 18, was located on the same side of the first (horizontal) axis together with *C. hartmaniorum* genotypes. The remaining three groups were located on the opposite side of the horizontal axis which explained more variance between the objects (37.8%) compared to the second axis (15.2% of the variance explained). The Dutch population 10 and the Polish population 15 were placed in the upper part of the vertical axis, in a considerable distance from the Estonian populations 17 and 19 (Fig. 6).

## Discussion

### Variability within *Carex hartmaniorum* populations

Analysis of the ISSR markers has shown the *C. hartmaniorum* populations to be more genetically homogenous than the populations of *C*. *buxbaumii*. Consequently, the microsatellite primers chosen proved less effective in detecting polymorphism and variability in the *C. hartmaniorum* populations. There are, however, grounds to continue studying the species’ genetic variability, particularly in view of the considerable morphological variation. It seems also purposeful to use combinations of other molecular methods, useful in exploring relationships between closely related taxa within the genus *Carex*, e.g., SSR markers, sequencing of the ITS nuclear ribosomal DNA region and the rbcL chloroplast region or more sensitive methods based on high-throughput sequencing technologies such as the Genotyping-by-Sequencing (GBS) or Next Generation Sequencing (NGS) (Rejzková et al., 2008; Korpelainen et al., 2010; Jiménez-Mejías et al., 2011; Jiménez-Mejías et al., 2012; Escudero et al., 2014).

The morphological variability in *C*. *hartmaniorum* observed in our study could be associated with environmental effects, and, to a lesser extent, with genetic factors. Phenotypic plasticity in plants is considered to be one of the ways plants respond morphologically and/or physiologically to environmental changes (Gratani, 2014). Variability of morphological characters observed in sedges could have resulted from habitat conditions, as demonstrated in the sections *Ceratotystis* Dumort. (e.g., Więcław & Podlasiński, 2013; Więcław, 2017) and *Phaestoglochin* Dumort. (e.g., Janyszek & Jagodzinski, 2009; Jagodziński et al., 2017).

The *C*. *hartmaniorum* population from the island of Terschelling (the Netherlands), the most morphologically and genetically divergent among the *C*. *hartmaniorum* populations studied, grows in a secondary dune valley slightly overblown with sand, with a strongly fluctuating water level (Kern, 1967; Westhoff & Ketner, 1967). The morphological distinctness of the specimens from that population (number 1) resulted primarily from differences in the utricle size, i.e., a generative character, most important in the identification of *Carex* taxa (Chater, 1980; Egorova, 1999). Generative characters in seed plants are, as a rule, less variable than the vegetative ones (e.g., Rakić et al., 2012; Preston, 2013), but both character groups are more or less closely associated with a taxon’s adaptation to local habitat conditions (e.g., Hereford, 2009; Raabová, Münzbergová & Fischer, 2011; Więcław, 2017). It is highly probable that the morphology of *C*. *hartmaniorum* was affected by the relatively nutrient-rich and specific coastal habitat of the island of Terschelling (Westhoff & Ketner, 1967). The phenotypic and genetic distinctness of the Dutch population is (probably) the result of geographic and habitat isolation; it forms the only population of this species in the Netherlands. Populations subjected to prolonged geographic and habitat isolation may diverge due to genetic drift, varying selection pressure, and mutations (Wang & Bradburd, 2014; Wanderley et al., 2018).

Another morphologically distinct population was the Armenian one; generally, the Armenian individuals were smaller than others, which most probably resulted from their adaptation to living in a mountain area (see Körner, 1999). The Armenian occurrences belonging to the populations of *C*. *hartmaniorum* represent the upper limit of the species’ altitude range (the site was located at 2,260 m a.s.l.). Lower temperatures in the mountains limit cell divisions and result in a smaller size of the plant (Körner et al., 1989). The limited plant growth at high altitudes may result from an abiotic stress caused, in addition to the lower temperature, by a low CO_2_ partial pressure and a high UV radiation (Körner, 2007), as well as a thin soil layer and hence a low nutrient availability (Huber et al., 2007). The occurrence of dwarf morphotypes in the mountains is fairly frequently observed in the genus *Carex* (e.g., Jiménez-Mejías et al., 2017; Więcław, 2017).

### Variability within *Carex buxbaumii* populations

The populations of *C*. *buxbaumii* were more variable than those of *C*. *hartmaniorum*, both morphologically and genetically. The microsatellite primers chosen for the ISSR polymorphism analysis, particularly the repeated nucleotide sequences (GA)_8_, effectively differentiated between the populations studied, as shown by the two multivariate statistical methods used (UPGMA and PCA).

The genetic distance-based grouping of the *C*. *buxbaumii* populations was generally similar (but not identical) to the grouping produced by the morphological data. The agreement between grouping with the DFA as well as the PCA and UPGMA was found between the Polish (11 and 13) and Estonian (17 and 19) populations. Their morphological characters were very similar, as were their genetic traits. Moreover, the DFA and PCA showed two populations (14 from Poland and 18 from Estonia) to distinctly differ from the remaining specimens. These populations showed the largest phenotypic and genetic differences with respect to the Dutch population (10). The relatively high genetic variability in the latter, compared to a fairly low genetic variability observed in the Polish and Estonian populations, could have resulted from a sampling effect (see Plant material and specimen collection).

The morphology of the specimens forming the Polish and Estonian populations (14 and 18) corresponds to the description of varieties *brevisquamosa* and *confusa*, with the utricle and glume size as diagnostic characters. According to Cajander (1935), utricles in the varieties *brevisquamosa* and *confusa* are usually smaller (3–3.5 mm) than those in the typical variety (3.0–4.5 mm, usually about 4.0 mm). The apex of the glume is usually acuminate or aristate with an awn; in the typical variety, the awn is by $ \frac{1}{4} $ to 2 times longer than the glume and distinctly exceeds the utricle, while in var. *brevisquamosa* and var. *confusa* it is by ^1^/_5_ to $ \frac{1}{2} $ times longer than the glume and is usually shorter (var. *brevisquamosa*) or slightly longer (var. *confusa*) than the utricle Cajander (1935). The Polish population (14) as well as the Estonian one (18) contained specimens morphologically close to both var. *brevisquamosa* and var. *confusa* as well as intermediate ones, but they all were distinctly different, morphologically and genetically, from the typical form. The morphological distinctness of the specimens from those populations was already visible during the fieldwork, especially in the case where they were growing close to one another. That was the case with two Polish populations: 13 (typical specimens) and 14 (specimens similar to var. *brevisquamosa* and var. *confusa*). Both populations were growing in a calcareous meadow, about 50 m from each other, which may suggest similar habitat conditions at the sites of occurrence. On the other hand, effects of (micro)habitat conditions on morphology of those specimens cannot be ruled out. In nature, intraspecific differences may be underlain by numerous mechanisms, including local adaptations, phenotypic plasticity, parental conditions, and selection (Violle et al., 2012). As shown by studies on the *C*. *flava* complex, periodic flooding, local desiccation, trampling, sun exposure or local edaphic conditions may lead to the emergence of different morphotypes (Więcław & Podlasiński, 2013; Więcław, 2017). In addition, the type of land use (grazing, mowing) may affect plant morphology as well (e.g., Meer van der et al., 2014; Kostrakiewicz-Gierałt, Stachurska-Swakoń & Towpasz, 2018). Generally, plants growing on pastures are usually smaller than plants found in abandoned and mown areas (e.g., Liu & Li, 2010). According to Sienkiewicz-Paderewska et al. (2019), *C*. *buxbaumii* growing on extensively mown sites with poorer light conditions employs a shade avoidance strategy (see Huber & Wiggermann, 1997) and develops higher culms as an advantage in the relatively strong competition for light.

Studies on plant genetic variability revealed a connection between the genetic variation within a species and its geographic range. Those species with a restricted geographic distribution usually show a lower level of genetic variability, compared to widely distributed species, due to effects of either directional selection promoting adaptation to local environments or random processes such as inbreeding, genetic bottlenecks, or drift acting in small populations (e.g., Gibson, Rice & Stucke, 2008; Levy et al., 2016). Therefore, the results of this study demonstrating a morphological and genetic distinctness of the Estonian and Polish populations should be carefully interpreted with relation to the putative infraspecific entities described by Cajander (1935). *Carex buxbaumii* has a wide distribution range, for which reason the distinctness of those populations may disappear when samples are collected from other geographic areas and populations from the entire range are compared.

In addition, it should be stressed that Lipnerová et al. (2013) consider section *Racemosae* as a product of polyploidy, and suggest a polyploid origin of *C*. *buxbaumii*. Lipnerová et al. (2013) demonstrated that a substantial increase in the chromosome count (mostly doubling) corresponded with a concomitant increase in the genome size in the sections *Clandestinae* and *Racemosae*; in the latter, the genome size and the chromosome counts in *C*. *buxbaumii* and *C*. *adelostoma* were almost twice those in *C*. *aterrima*, *C*. *parviflora*, *C*. *atrata*, *C*. *norvegica* and *C*. *hartmaniorum*. Moreover, the species were referred to as having different cytotypes; mainly 2n = 100, 102, 106 for *C*. *buxbaumii*, and 2n = 52, 68 for *C*. *hartmaniorum* (see Więcław, Kalinka & Koopman, 2020). Generally, polyploidy in *Carex* is relatively rare and is fairly difficult to find due to chromosomal traits (i.e., holocentric chromosomes) (Hipp, Rothrock & Roalson, 2009). In autopolyploidy, it is expected that a tetraploid species will have twice as many chromosomes as the initial diploid species, and the genome size in the former will be twice of that in the latter. However, if the polyploidy event is relatively ancient evolutionarily, this direct relationship is most often blurred by a DNA sequence loss/acquisition, aneupolyploidy or other phenomena occurring during evolution (Roalson, McCubbin & Whitkus, 2007).

Polyploidy plays a prominent role in genetic variability of most plants. Polyploids are regarded as being more plastic than diploids (e.g., Shi et al., 2015; McIntyre & Strauss, 2017), hence presumably a higher variability in the *C*. *buxbaumii* populations than in the *C*. *hartmaniorum* ones we studied. The evolutionary success of polyploids is considered to be associated with new phenotypes which exceed the range of their diploid progenitors (Ramsey & Schemske, 2002).

In alloploid organisms emerging as a result of interspecific hybridization, the phenotypic and genetic variability of the hybrid population may be much higher than that of the parental populations, including their combined level (transgressive segregation). Hybridization, i.e., the emergence of a taxon resulting from cross-breeding of different species, and introgression, i.e., the movement of genes from one species into the gene pool of another by back-crossing an interspecific hybrid with one of its parents, are common in *Carex* (Korpelainen et al., 2010; Escudero et al., 2014). These are most likely the major causes of the high morphological variability and unclear taxonomic status among certain sections of *Carex*, including the *Racemosae.* It is highly probable that introgression occurs between the two species studied, *C*. *buxbaumii* and *C*. *hartmaniorum*, and is manifested as the flow of some genes, including those of adaptive significance, due to hybridization.

It is probable that the lineage to which *C*. *hartmaniorum* belongs (i.e., its ancestor) or the latter itself have been involved in the formation of *C*. *buxbaumii*. Results of our study point to a considerable genetic affinity between the two sedge species, as evidenced by the mean Si of 0.619. In addition, some populations of *C*. *buxbaumii* were genetically more similar to *C*. *hartmaniorum* than to conspecific populations. The UPGMA dendrogram constructed based on the genetic similarity metric grouped most *C. buxbaumii* populations together, in a joint major grouping, with *C. hartmaniorum*. Although the two sedges were assigned to two separate sub-groups (a and b), this may be taken as a joint origin of some alleles. Unfortunately, our study, by focussing primarily on the assessment of morphological and genetic variability of the two sedge species, does not lend itself to resolving this question, and the consideration presented above are exclusively hypothetical. Resolving the question would call for continuation and a detailed phylogenetic revision based on, inter alia, high-throughput sequencing technologies and analyses of phylogenetic trees. The challenges associated with such approaches involve, among others, finding genetic markers with enough phylogenetic signal and discordance among gene trees inferred from individual markers, which may differ significantly from the species or population tree. Such discordance may originate from hybridization among fully differentiated species with subsequent fixation of loci or incomplete random sorting of alleles due to short intervals between divergence events (ILS) (Escudero et al., 2014).

## Conclusions

The populations of *C*. *buxbaumii* studied were, morphologically and genetically, more heterogeneous than the populations of *C*. *hartmaniorum*. Two populations of *C*. *buxbaumii* (from Poland and Estonia) were separated from the remaining populations, as their individuals showed shorter utricles and glumes, compared to the typical specimens of *C*. *buxbaumii*, and corresponded with the morphology of putative infraspecific entities described by Cajander (1935). However, the distribution range of *C*. *buxbaumii* is wide, therefore the differences between these populations may become blurred when sampling is extended to other geographic areas and when populations from throughout the range are examined.

The taxonomic status of the putative infraspecific taxa within *C. buxbaumii* requires further studies throughout the distribution range of *C*. *buxbaumii*, including a chromosome count and genome size, particularly as the variability in *C*. *buxbaumii* may be a result of the species’ polyploid origin.

The results obtained in this study motivate us to continue the study and to take a more comprehensive approach to the variability observed in the sedges investigated. Habitat-oriented, morphological and genetic analyses, the latter including the chromosome number and combinations of various genetic techniques, should not only expand the knowledge on the variability within *C*. *hartmaniorum* and *C*. *buxbaumii*, but ought to be used in a more general discussion on polyploidy and its importance in the evolution of plants.

##  Supplemental Information

10.7717/peerj.11372/supp-1 Supplemental Information 1 Raw data for evaluation of morphological variability of the analyzed populations

10.7717/peerj.11372/supp-2 Supplemental Information 2 Raw data for the evaluation of genetic variability for all populations analyzed in this study: a) values of genetic similarity index (Si) and b) data parameters for calculating the values of molecular variance (AMOVA) in the GenAIEx 6.5 software

10.7717/peerj.11372/supp-3 Supplemental Information 3 Results of U Mann–Whitney test, showing difference between *Carex buxbaumii* and *C*. *hartmaniorum*; significance level, * *p* ≤ 0.05

10.7717/peerj.11372/supp-4 Supplemental Information 4 Morphological characters of *Carex buxbaumii* and *C. hartmaniorum.* Me, median; range, minimum and maximum values; IQR, interquartile range; V, coefficient of variation.

10.7717/peerj.11372/supp-5 Supplemental Information 5 Results of Kruskal-Wallis test and post-hoc Dunn’s multiple comparisons test, showing significance of differences in morphological characters of *Carex buxbaumii* populations p, significance level; significant differences ( *p* ≤ 0.05) have been marked with bold; CH, Culm height; LW, Leaf width; BL, Bract length; IL, Inflorescence length; NS, Number of female spikes; USL, Uppermost spike length; USW, Uppermost spike width; LSL, Lowest spike length; LSW, Lowest spike width; UL, Utricle length; UBL, Utricle beak length; GL, Glume length; 10, 11, 13, 14, …, number of *C*. *buxbaumii* populations (see Table 1).

10.7717/peerj.11372/supp-6 Supplemental Information 6 Results of Kruskal-Wallis test and post-hoc Dunn’s multiple comparisons test, showing significance of differences in morphological characters of *Carex hartmaniorum* populations p, significance level; significant differences ( *p* ≤ 0.05) have been marked with bold; CH, Culm height; LW, Leaf width; BL - Bract length; IL, Inflorescence length; NS, Number of female spikes; USL, Uppermost spike length; USW, Uppermost spike width; LSL,- Lowest spike length; LSW, Lowest spike width; UL, Utricle length; UBL, Utricle beak length; GL, Glume length; 1, 2, 4, 5, …, number of *C*. *hartmaniorum* populations (see Table 1).

10.7717/peerj.11372/supp-7 Supplemental Information 7 Comparison of morphological vegetative characters of *Carex buxbaumii* and *C*. *hartmaniorum* populations Large boxes indicate 25–75% of the interquartile ranges of values, small black boxes—the medians, circle—outlier values, asterisks—extreme values. The populations are numbered according to Table 1.

10.7717/peerj.11372/supp-8 Supplemental Information 8 Comparison of size spikes of *Carex buxbaumii* and *C*. *hartmaniorum* populations Large boxes indicate 25–75% of the interquartile ranges of values, small black boxes—the medians, circle—outlier values, asterisks—extreme values. The populations are numbered according to Table 1.

10.7717/peerj.11372/supp-9 Supplemental Information 9 Comparison of size utricles and beaks of* Carex buxbaumii* and *C*. *hartmaniorum* populations Large boxes indicate 25–75% of the interquartile ranges of values, small black boxes—the medians, circle—outlier values, asterisks—extreme values. The populations are numbered according to Table 1.

## Additional Information and Declarations

### Field Study Permissions

The following information was supplied relating to field study approvals (i.e., approving body and any reference numbers): No official permits for our research were required because (i) all field research was conducted outside protected areas, on sites where sedges were abundant, (ii) the study did not concern protected taxa with the exception of *Carex buxbaumii* in Poland. However, we collected *Carex buxbaumii* in Poland in 2013, when it was not under legal protection yet (Regulation of the Minister of the Environment from 2012). This species has been protected in Poland since 2014 (Regulation of the Minister of the Environment from 2014). In Poland, we conducted research outside protected areas. According to The Nature Conservation Act of April 16, 2004, Art. 15.1 point 24 it is forbidden to conduct scientific research only in national parks and nature reserves. In the Netherlands, according to https://wetten.overheid.nl/BWBR0037552/2017-01-01#Opschrift Article 3.5 5 and 3.10 1c it is forbidden to pick and collect the plants mentioned in Appendix IVb (*Carex hartmaniorum* [Kleine Knotszegge] is not included in this appendix).

### Data Availability

The following information was supplied regarding data availability: The raw data for evaluation of genetic variability and raw measurements for evaluation of morphological variability are available in the Supplementary Files.
