# Supplementary material for: Morphological variability and genetic diversity in Carex buxbaumii and Carex hartmaniorum (Cyperaceae) populations
Source: PeerJ. 2021 May 11;9:e11372. doi: 10.7717/peerj.11372 (PMC8121068; doi:10.7717/peerj.11372)
Supplement: Supplemental Information 3 [file peerj-09-11372-s003.docx]

Table S1:

Results of U Mann-Whitney test, showing difference between *Carex buxbaumii* and *C*. *hartmaniorum*; significance level, *p ≤ 0.05.

| Characters | U Mann-Whitney test | |
| --- | --- | --- |
|  | *Z* | *p* |
| Culm height | 0.027 | 0.9787 |
| Leaf width | -2.433 | 0.0149* |
| Bract length | -0.829 | 0.4071 |
| Inﬂorescence length | 6,372 | 0.0000* |
| Number of female spikes | 9.96 | 0.0000* |
| Uppermost spike length | 6.485 | 0.0000* |
| Uppermost spike width | -12.746 | 0.0000* |
| Lowest spike length | 10.959 | 0.0000* |
| Lowest spike width | -9.905 | 0.0000* |
| Utricle length | -14.704 | 0.0000* |
| Utricle beak length | -1.644 | 0.1001 |
| Glume length | -13.099 | 0.0000* |
