# Supplementary material for: Morphological variability and genetic diversity in Carex buxbaumii and Carex hartmaniorum (Cyperaceae) populations
Source: PeerJ. 2021 May 11;9:e11372. doi: 10.7717/peerj.11372 (PMC8121068; doi:10.7717/peerj.11372)
Supplement: Supplemental Information 4 — Me, median; range, minimum and maximum values; IQR, interquartile range; V, coefficient of variation. [file peerj-09-11372-s004.docx]

Table S2:

Morphological characters of *Carex buxbaumii* and *C. hartmaniorum*; Me – median; range – minimum and maximum values; IQR – interquartile range; V – coefﬁcient of variation.

| Characters | *Carex buxbaumii* (n = 160) | | | | *Carex hartmaniorum* (n = 140) | | | |
| --- | --- | --- | --- | --- | --- | --- | --- | --- |
|  | *Me* | Range | *IQR* | *V* | *Me* | Range | *IQR* | *V* |
| Culm height | 57.5 | 29.2-88.8 | 18.85 | 20.6 | 59.8 | 27.4-87.2 | 19.35 | 21.3 |
| Leaf width | 0.28 | 0.2-0.4 | 0.05 | 14.3 | 0.27 | 0.2-0.4 | 0.05 | 13.0 |
| Bract length | 6.11 | 3.0-23.6 | 3.66 | 45.0 | 5.91 | 2.4-14.9 | 2.97 | 39.9 |
| Inﬂorescence length | 5.39 | 3.1-11.9 | 2.06 | 30.3 | 6.90 | 3.8-15.8 | 2,19 | 31.2 |
| Number of female spikes | 4.00 | 3.0-5.0 | 1.00 | 14.9 | 4.00 | 3.0-7.0 | 1.00 | 14.9 |
| Uppermost spike length | 2.01 | 1.3-2.7 | 0.34 | 11.9 | 2.28 | 1.4-3.2 | 0.48 | 16.5 |
| Uppermost spike width | 0.84 | 0.6-1.1 | 0.15 | 12.9 | 0.64 | 0.4-0.85 | 0.10 | 12.0 |
| Lowest spike length | 1.59 | 1.0-2.6 | 0.44 | 18.5 | 2.28 | 1.0-3.4 | 0.67 | 21.9 |
| Lowest spike width | 0.68 | 0.35-1.1 | 0.20 | 18.9 | 0.52 | 0.3-0.75 | 0.13 | 15.9 |
| Utricle length | 3.64 | 3.1-4.2 | 0.29 | 5.6 | 2.84 | 2.5-3.45 | 0.21 | 6.9 |
| Utricle beak length | 0.19 | 0.1-0.3 | 0.05 | 18.8 | 0.18 | 0.15-0.25 | 0.03 | 12.3 |
| Glume length | 4.58 | 3.05-6.6 | 0.98 | 15.6 | 3.34 | 3.0-4.55 | 0.34 | 8.1 |
