# Supplementary material for: Morphological variability and genetic diversity in Carex buxbaumii and Carex hartmaniorum (Cyperaceae) populations
Source: PeerJ. 2021 May 11;9:e11372. doi: 10.7717/peerj.11372 (PMC8121068; doi:10.7717/peerj.11372)
Supplement: Supplemental Information 5 — p, significance level; significant differences ( p ≤ 0.05) have been marked with bold; CH, Culm height; LW, Leaf width; BL, Bract length; IL, Inflorescence length; NS, Number of female spikes; USL, Uppermost spike length; USW, Uppermost spike width; LSL, Lowest spike length; LSW, Lowest spike width; UL, Utricle length; UBL, Utricle beak length; GL, Glume length; 10, 11, 13, 14, …, number of C. buxbaumii populations (see Table 1). [file peerj-09-11372-s005.docx]

Table S3:

Results of Kruskal-Wallis test and post-hoc Dunn’s multiple comparisons test, showing signiﬁcance of differences in morphological characters of *Carex buxbaumii* populations. p – significance level; significant differences (p ≤ 0.05) have been marked with bold.

| Traits | *Carex buxbaumii* | | | | | | | | | | | | | | | | | | | | | | | | | | | | | |
| --- | --- | --- | --- | --- | --- | --- | --- | --- | --- | --- | --- | --- | --- | --- | --- | --- | --- | --- | --- | --- | --- | --- | --- | --- | --- | --- | --- | --- | --- | --- |
|  | Kruskal–Wallis test | | Dunn's multiple comparisons test | | | | | | | | | | | | | | | | | | | | | | | | | | | |
|  |  |  | 10-11 | 10-13 | 10-14 | 10-16 | 10-17 | 10-18 | 10-19 | 11-13 | 11-14 | 11-16 | 11-17 | 11-18 | 11-19 | 13-14 | 13-16 | 13-17 | 13-18 | 13-19 | 14-16 | 14-17 | 14-18 | 14-19 | 16-17 | 16-18 | 16-19 | 17-18 | 17-19 | 18-19 |
|  | *H* | *p* | *p* | *p* | *p* | *p* | *p* | *p* | *p* | *p* | *p* | *p* | *p* | *p* | *p* | *p* | *p* | *p* | *p* | *p* | *p* | *p* | *p* | *p* | *p* | *p* | *p* | *p* | *p* | *p* |
| CH | 98.46 | 0.00 | 0.27 | 1.00 | **0.02** | **0.00** | 1.00 | **0.01** | 1.00 | 1.00 | **0.00** | **0.00** | 0.38 | **0.00** | 1.00 | **0.00** | **0.00** | 1.00 | **0.00** | 1.00 | 1.00 | **0.02** | 1.00 | **0.00** | **0.00** | 1.00 | **0.00** | **0.00** | 1.00 | 1.00 |
| LW | 68.42 | 0.00 | 1.00 | 1.00 | **0.00** | **0.04** | 0.92 | **0.00** | 0.81 | 1.00 | **0.00** | **0.00** | 0.08 | **0.00** | 0.07 | **0.00** | **0.00** | 0.13 | **0.00** | 0.12 | 1.00 | 0.15 | 1.00 | 0.17 | 1.00 | 1.00 | 1.00 | 1.00 | 1.00 | 1.00 |
| BL | 32.24 | 0.00 | 1.00 | 1.00 | 1.00 | 0.09 | 0.12 | 1.00 | 1.00 | 1.00 | 1.00 | **0.01** | **0.01** | 1.00 | 1.00 | 0.58 | **0.00** | **0.00** | 1.00 | 1.00 | 1.00 | 1.00 | 1.00 | 1.00 | 1.00 | 0.06 | 0.46 | 0.08 | 0.53 | 1.00 |
| IL | 65.39 | 0.00 | 1.00 | 1.00 | **0.00** | **0.00** | **0.00** | **0.02** | 0.06 | 1.00 | **0.02** | **0.00** | **0.00** | 0.43 | 0.87 | **0.00** | **0.00** | **0.00** | 0.15 | 0.33 | 1.00 | 1.00 | 1.00 | 1.00 | 1.00 | 0.25 | 0.11 | 1.00 | 1.00 | 1.00 |
| NS | 24.93 | 0.00 | 1.00 | 1.00 | 1.00 | 0.82 | 1.00 | 1.00 | 1.00 | 1.00 | 0.61 | 0.19 | 0.45 | 1.00 | 1.00 | 1.00 | 0.82 | 1.00 | 1.00 | 1.00 | 1.00 | 1.00 | 0.29 | 1.00 | 1.00 | 0.08 | 1.00 | 0.21 | 1.00 | 1.00 |
| USL | 25.78 | 0.00 | 1.00 | 1.00 | 1.00 | 1.00 | 1.00 | 0.28 | 1.00 | 1.00 | 0.08 | 1.00 | 1.00 | 1.00 | 1.00 | 1.00 | 1.00 | 1.00 | 0.44 | 1.00 | 1.00 | 1.00 | **0.00** | 0.44 | 1.00 | **0.03** | 1.00 | **0.01** | 1.00 | 1.00 |
| USW | 94.51 | 0.00 | 1.00 | 1.00 | **0.00** | **0.00** | **0.00** | **0.00** | 0.19 | 1.00 | **0.00** | 0.44 | 1.00 | **0.00** | 1.00 | **0.00** | 0.14 | 1.00 | **0.00** | 1.00 | 0.24 | **0.01** | 1.00 | **0.00** | 1.00 | 0.09 | 1.00 | **0.00** | 1.00 | **0.00** |
| LSL | 42.71 | 0.00 | 1.00 | 1.00 | 1.00 | 1.00 | 1.00 | **0.00** | 1.00 | 0.15 | 1.00 | 0.23 | 1.00 | 0.26 | 1.00 | 0.29 | 1.00 | 1.00 | **0.00** | **0.03** | 0.46 | 1.00 | 0.13 | 1.00 | 1.00 | **0.00** | **0.05** | **0.02** | 1.00 | 1.00 |
| LSW | 87.25 | 0.00 | 1.00 | 1.00 | **0.00** | 0.89 | **0.03** | **0.00** | **0.02** | 1.00 | **0.00** | 1.00 | 1.00 | **0.00** | 1.00 | **0.00** | 0.36 | **0.01** | **0.00** | **0.01** | **0.00** | **0.05** | 1.00 | 0.07 | 1.00 | **0.00** | 1.00 | 0.17 | 1.00 | 0.24 |
| UL | 57.42 | 0.00 | 1.00 | 1.00 | 0.77 | 1.00 | 0.07 | **0.00** | 1.00 | 1.00 | 0.04 | 1.00 | **0.00** | **0.00** | 1.00 | 0.88 | 1.00 | 0.08 | **0.00** | 1.00 | **0.00** | 1.00 | 1.00 | 1.00 | **0.00** | **0.00** | 0.32 | 1.00 | 0.47 | **0.03** |
| UBL | 48.20 | 0.00 | 1.00 | 1.00 | 1.00 | 1.00 | 0.45 | 0.78 | 1.00 | **0.00** | 1.00 | **0.00** | **0.00** | 1.00 | **0.01** | 0.22 | 1.00 | 1.00 | **0.00** | 1.00 | 0.11 | **0.02** | 1.00 | 0.56 | 1.00 | **0.00** | 1.000 | **0.00** | 1.00 | **0.01** |
| GL | 113.41 | 0.00 | 1.00 | 1.00 | **0.00** | 0.08 | **0.01** | **0.00** | **0.00** | 1.00 | **0.00** | 1.00 | 0.56 | **0.00** | 0.20 | **0.00** | 0.40 | **0.04** | **0.00** | **0.01** | **0.00** | **0.04** | 1.00 | 0.12 | 1.00 | **0.00** | 1.00 | **0.00** | 1.00 | **0.01** |

Explanations: CH - Culm height; LW - Leaf width; BL - Bract length; IL - Inﬂorescence length; NS - Number of female spikes; USL - Uppermost spike length; USW - Uppermost spike width; LSL - Lowest spike length; LSW - Lowest spike width; UL - Utricle length; UBL - Utricle beak length; GL - Glume length; 10, 11, 13, 14, ... - number of *C*. *buxbaumii* populations (see Table 1).
