# Supplementary material for: Morphological variability and genetic diversity in Carex buxbaumii and Carex hartmaniorum (Cyperaceae) populations
Source: PeerJ. 2021 May 11;9:e11372. doi: 10.7717/peerj.11372 (PMC8121068; doi:10.7717/peerj.11372)
Supplement: Supplemental Information 6 — p, significance level; significant differences ( p ≤ 0.05) have been marked with bold; CH, Culm height; LW, Leaf width; BL - Bract length; IL, Inflorescence length; NS, Number of female spikes; USL, Uppermost spike length; USW, Uppermost spike width; LSL,- Lowest spike length; LSW, Lowest spike width; UL, Utricle length; UBL, Utricle beak length; GL, Glume length; 1, 2, 4, 5, …, number of C. hartmaniorum populations (see Table 1). [file peerj-09-11372-s006.docx]

Table S4:

Results of Kruskal-Wallis test and post-hoc Dunn’s multiple comparisons test, showing signiﬁcance of differences in morphological characters of *Carex hartmaniorum* populations. p – significance level; significant differences (p ≤ 0.05) have been marked with bold.

| Traits | *Carex hartmaniorum* | | | | | | | | | | | | | | | | | | | | | | |
| --- | --- | --- | --- | --- | --- | --- | --- | --- | --- | --- | --- | --- | --- | --- | --- | --- | --- | --- | --- | --- | --- | --- | --- |
|  | Kruskal–Wallis test | | Dunn's multiple comparisons test | | | | | | | | | | | | | | | | | | | | |
|  |  |  | 1-2 | 1-4 | 1-5 | 1-7 | 1-8 | 1-9 | 2-4 | 2-5 | 2-7 | 2-8 | 2-9 | 4-5 | 4-7 | 4-8 | 4-9 | 5-7 | 5-8 | 5-9 | 7-8 | 7-9 | 8-9 |
|  | *H* | *p* | *p* | *p* | *p* | *p* | *p* | *p* | *p* | *p* | *p* | *p* | *p* | *p* | *p* | *p* | *p* | *p* | *p* | *p* | *p* | *p* | *p* |
| CH | 92.28 | 0.00 | 1.00 | 0.09 | **0.00** | 0.31 | **0.00** | 0.21 | 0.06 | **0.00** | 0.23 | **0.00** | 0.29 | 1.00 | 1.00 | 0.69 | **0.00** | 0.49 | 1.00 | **0.00** | 0.23 | **0.00** | **0.00** |
| LW | 32.12 | 0.00 | 1.00 | **0.01** | 1.00 | 0.77 | 0.14 | 1.00 | 0.14 | 1.00 | 1.00 | 0.97 | 1.00 | **0.00** | 1.00 | 1.00 | **0.00** | 0.34 | 0.05 | 1.00 | 1.00 | 0.09 | **0.01** |
| BL | 24.52 | 0.00 | 1.00 | 1.00 | **0.01** | 1.00 | 0.27 | 0.09 | 1.00 | 0.42 | 1.00 | 1.00 | 1.00 | **0.00** | 1.00 | 0.14 | **0.05** | 0.53 | 1.00 | 1.00 | 1.00 | 1.00 | 1.00 |
| IL | 43.54 | 0.00 | 0.11 | 1.00 | **0.00** | 1.00 | **0.00** | 1.00 | 0.13 | 1.00 | 1.00 | 0.95 | 1.00 | **0.00** | 1.00 | **0.00** | 1.00 | 0.08 | 1.00 | 0.36 | **0.02** | 1.00 | 0.10 |
| NS | 28.09 | 0.00 | 1.00 | 1.00 | 1.00 | 0.09 | 1.00 | **0.02** | 1.00 | 1.00 | 1.00 | 1.00 | 0.67 | 1.00 | **0.03** | 0.69 | **0.00** | 1.00 | 1.00 | 0.47 | 1.00 | 1.00 | 1.00 |
| USL | 62.48 | 0.00 | 1.00 | 1.00 | 1.00 | 1.00 | **0.00** | **0.01** | 1.00 | 1.00 | 1.00 | **0.00** | **0.00** | 1.00 | 1.00 | **0.00** | **0.02** | 1.00 | 0.06 | **0.00** | 0.21 | **0.00** | **0.00** |
| USW | 49.34 | 0.00 | 0.39 | **0.00** | **0.00** | 0.06 | **0.00** | 1.00 | 0.67 | **0.01** | 1.00 | 0.65 | 1.00 | 1.00 | 1.00 | 1.00 | **0.04** | 0.09 | 1.00 | **0.00** | 1.00 | 1.00 | **0.04** |
| LSL | 60.85 | 0.00 | **0.00** | 1.00 | **0.04** | 1.00 | **0.00** | 0.71 | 0.33 | 1.00 | 0.34 | 1.00 | **0.00** | 1.00 | 1.00 | 0.17 | **0.01** | 1.00 | 1.00 | **0.00** | 0.18 | **0.01** | **0.00** |
| LSW | 60.73 | 0.00 | **0.03** | 0.53 | **0.00** | **0.00** | **0.00** | **0.00** | 1.00 | **0.01** | 0.53 | 1.00 | 1.00 | **0.00** | **0.03** | 0.08 | 1.00 | 1.00 | 1.00 | 0.08 | 1.00 | 1.00 | 1.00 |
| UL | 65.79 | 0.00 | **0.00** | **0.00** | **0.00** | **0.00** | **0.00** | **0.00** | **0.01** | 1.00 | 1.00 | 0.21 | 1.00 | 0.19 | 0.07 | 1.00 | 0.56 | 1.00 | 1.00 | 1.00 | 1.00 | 1.00 | 1.00 |
| UBL | 34.61 | 0.00 | 1.00 | 0.38 | 0.05 | **0.00** | 0.06 | **0.00** | 0.38 | 0.05 | **0.00** | 0.06 | **0.00** | 1.00 | 1.00 | 1.00 | 1.00 | 1.00 | 1.00 | 1.00 | 1.00 | 1.00 | 1.00 |
| GL | 41.26 | 0.00 | 1.00 | **0.00** | 0.56 | **0.01** | **0.00** | 0.34 | 0.15 | 1.00 | 1.00 | **0.01** | 1.00 | 0.15 | 1.00 | 1.00 | 0.27 | 1.00 | **0.03** | 1.00 | 1.00 | 1.00 | 0.06 |

Explanations: CH - Culm height; LW - Leaf width; BL - Bract length; IL - Inﬂorescence length; NS - Number of female spikes; USL - Uppermost spike length; USW - Uppermost spike width; LSL - Lowest spike length; LSW - Lowest spike width; UL - Utricle length; UBL - Utricle beak length; GL - Glume length; 1, 2, 4, 5, ... - number of *C*. *hartmaniorum* populations (see Table 1).
