# Supplementary figures and images for: Morphological variability and genetic diversity in Carex buxbaumii and Carex hartmaniorum (Cyperaceae) populations
Source: PeerJ. 2021 May 11;9:e11372. doi: 10.7717/peerj.11372 (PMC8121068; doi:10.7717/peerj.11372)

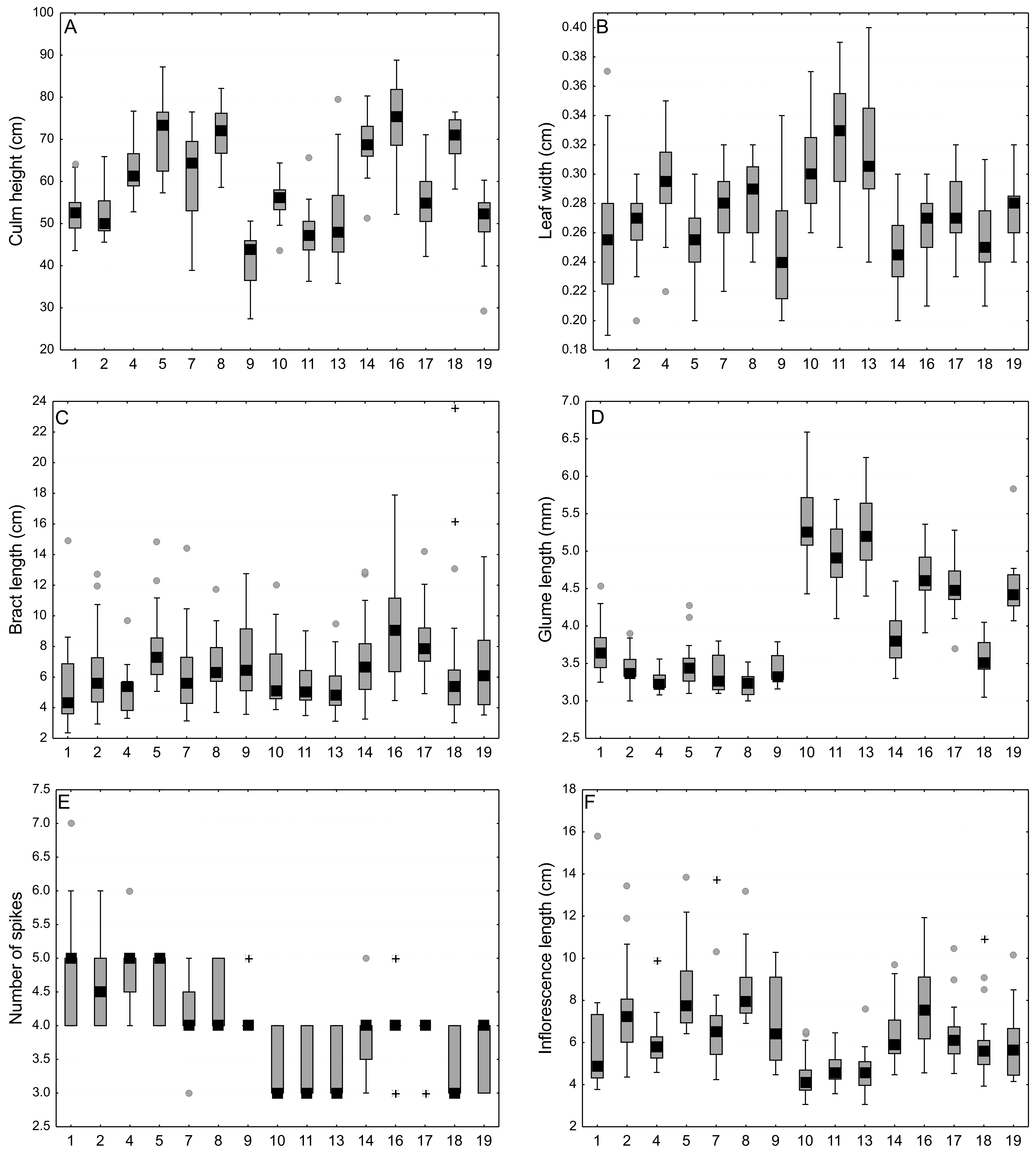

Supplement: Supplemental Information 7 — Large boxes indicate 25–75% of the interquartile ranges of values, small black boxes—the medians, circle—outlier values, asterisks—extreme values. The populations are numbered according to Table 1. [file peerj-09-11372-s007.png]

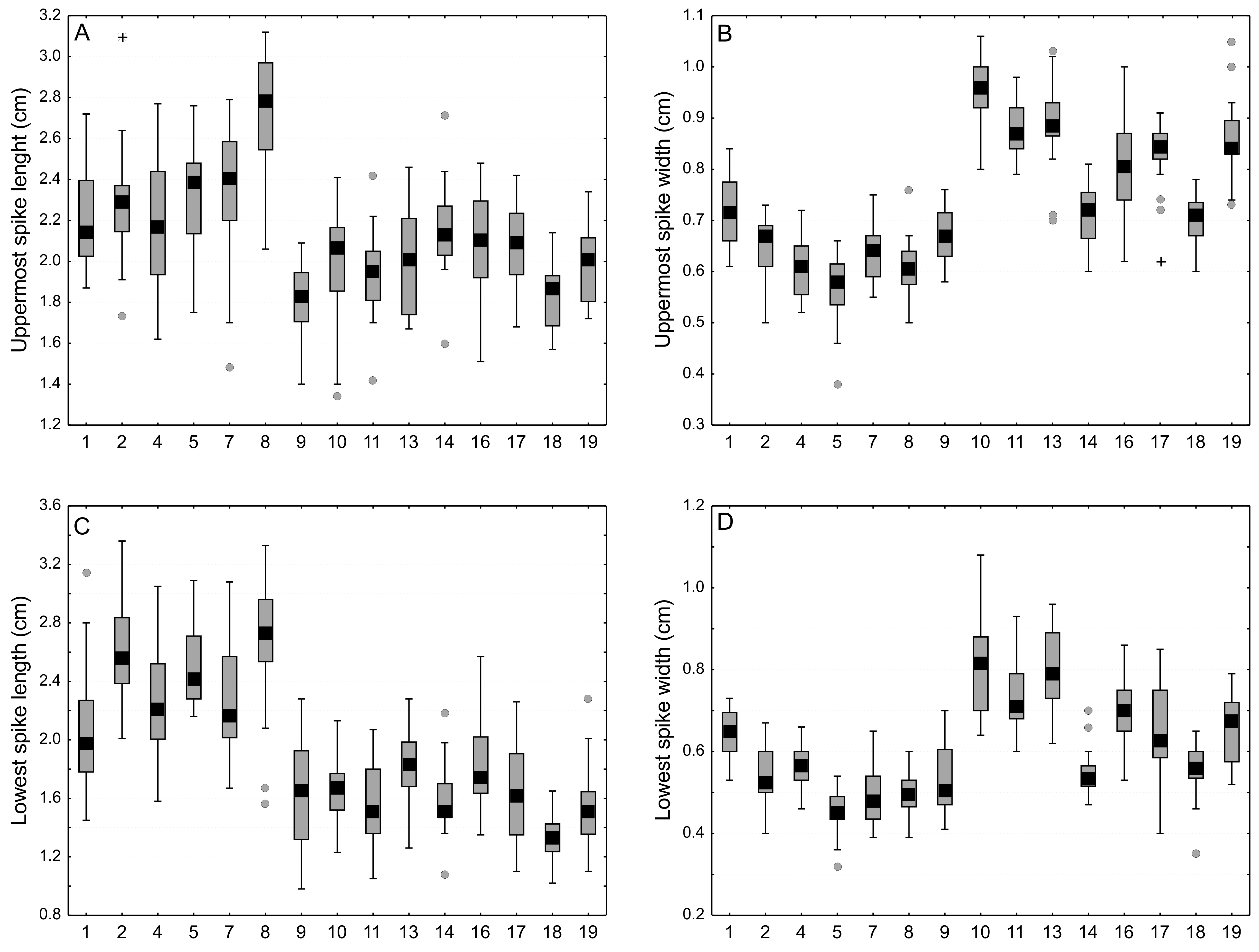

Supplement: Supplemental Information 8 — Large boxes indicate 25–75% of the interquartile ranges of values, small black boxes—the medians, circle—outlier values, asterisks—extreme values. The populations are numbered according to Table 1. [file peerj-09-11372-s008.png]

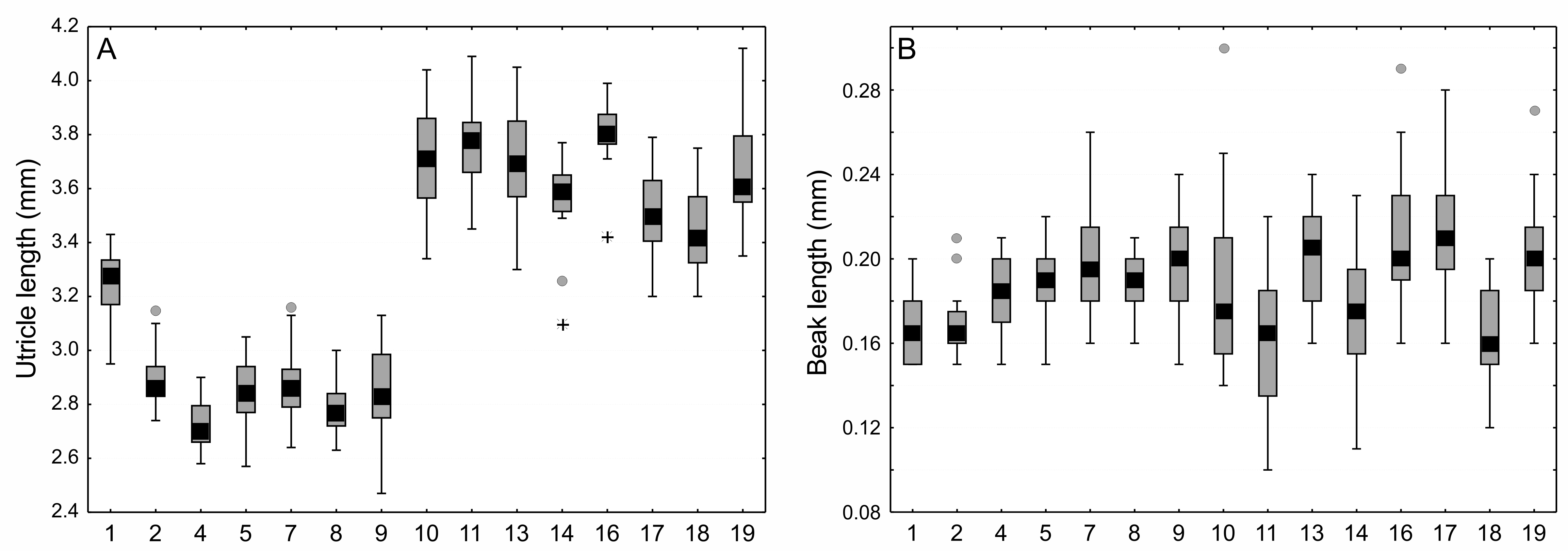

Supplement: Supplemental Information 9 — Large boxes indicate 25–75% of the interquartile ranges of values, small black boxes—the medians, circle—outlier values, asterisks—extreme values. The populations are numbered according to Table 1. [file peerj-09-11372-s009.png]
